# “In Dreams Begin Responsibilities”

**DOI:** 10.3201/eid1404.042008

**Published:** 2008-04

**Authors:** Polyxeni Potter

**Affiliations:** * Centers for Disease Control and Prevention, Atlanta, Georgia, USA

**Keywords:** Art and science, emerging infectious diseases, zoonotic diseases, Moschophoros, kouri and kores, marble sculpture, archaic sculpture, ancient Greek religious rituals, about the cover

**Figure:**
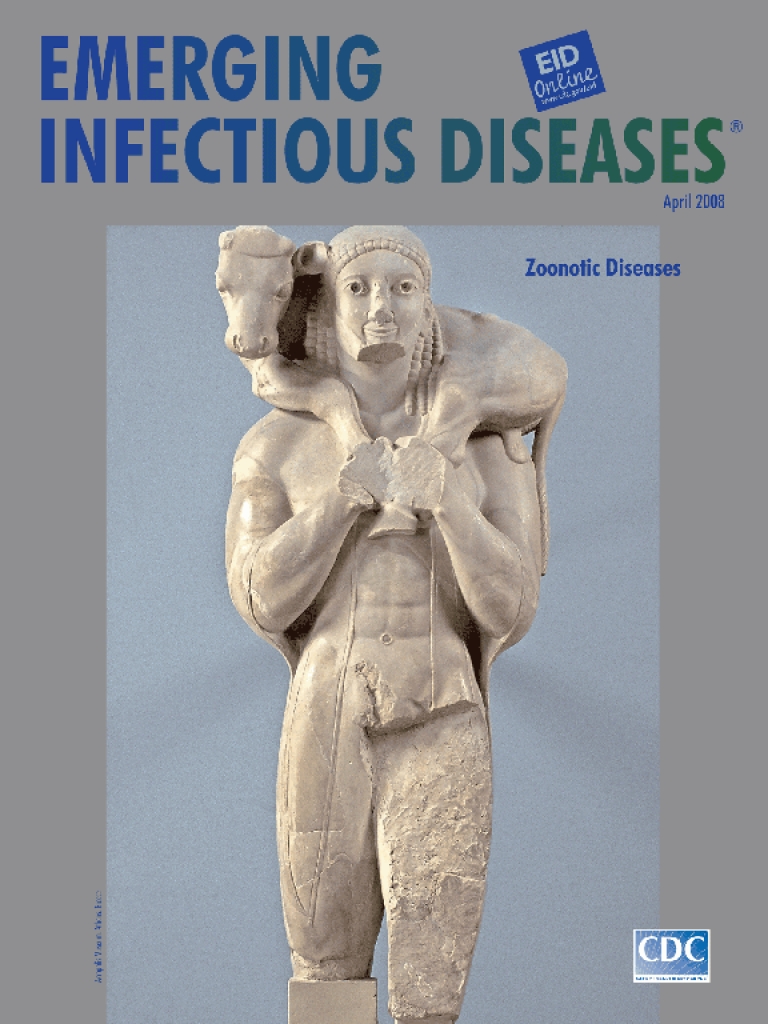
**Moschophoros (Calf-Bearer) attributed to Phaidimos. Statue of the patriot Romvos offering sacrificial calf to Athena.** c. 570 bce. Marble. Height 165 cm. No. 624 Acropolis Museum, Athens, Greece

—William Butler Yeats

“I woke with this marble head in my hands; / It exhausts my elbows and I don’t know where to put it down. / It was falling into the dream as I was coming out of the dream / So our life became one and it will be very difficult for it to separate again,” wrote George Seferis about his relationship with art from antiquity (*1*). Traversing the edges of time has long been the domain of artists and poets, who view history as a continuous process not to be fragmented and labeled “ancient” as if somehow interrupted or expired (*2*).

“The art of [marble] sculpture is much older than that of painting or bronze statuary,” wrote Pliny the Elder (23–79 ce) (*3*). Early sculptors worked on marble with point chisels, punches, and stone abrasives. Repeated vertical blows shattered crystals deep into the stone, altering the outer gloss. Because statues were painted, the opaque surface benefited pigment application. On the Acropolis, the first marble statues appeared more than two thousand years ago (*4*). They were votives, mostly maidens called *kores* but also young men, *kouri*. Some were inscribed with the names of artists; others, with dedications. They represented the donor or a deity, a renowned athlete, or the deceased if intended for a gravesite. They dominated art of the archaic period (750–500 bce). Thousands have been excavated from various sites.

Neither gods nor mortals, *kores* and *kouri* embodied physical perfection accessible to both. They were free-standing, the earliest such examples of large stone images of the human form in the history of art (*5*). Their arms were separated from the torso, the legs from each other. Tense and filled with life, they had various faces and expressions, their individuality foreshadowing portraiture. Their large eyes stared directly ahead, and they were injected with emotion, the stylized “archaic smile,” signifying not happiness but emerging humanity. They wore flowing garments, carefully delineated, and appeared refreshed and carefree, as if suddenly become aware of themselves.

The best-known of these figures, Moschophoros (calf-bearer), on this month’s cover, represented the donor, a nobleman named Romvos as inscribed on the base. The figure, found in fragments on the grounds of the Acropolis near the sanctuary of the Temple of Athena, has none of the masklike quality of earlier *kouri*. Though he has their usual left-foot-forward stance and stylized tufted hair, Moschophoros is not a youth but a mature man with a beard. His fitted cloak was likely painted in vivid colors as were the lips and hair. “The hollow eyes ... once held inlays of semi-precious stones (mother-of-pearl, gray agate, and lapis lazuli) that would have given the face a strikingly realistic appearance” (*6*).

Romvos is carrying an animal for sacrifice on the altar of Goddess Athena, a formidable fixture of the Hellenic pantheon known for its temperamental deities and countless demigods and their descendents. Their origins and relationships with humans were fodder for myths and art through sculpture and elaborate iconography. Gods gave gifts and favors. Humans offered votives as thanks, atonement, entreaty, or worship.

Sacrifice (from sacrificium [sacred] + facere [make] = to make sacred) was a central part of religious practice during festivals and feast days. The ritual was performed in well-defined space within a temple sanctuary. Some feasts were Pan-Hellenic and included processions and athletic competitions. “There are sanctuaries of Hermes Kriophoros,” wrote Pausanias, describing the city of Tanagra. “... Hermes averted a pestilence from the city by carrying a ram round the walls; to commemorate this Calamis made an image of Hermes carrying a ram upon his shoulders. Whichever of the youths is judged to be the most handsome goes round the walls at the feast carrying a lamb on his shoulders” (*7*).

Homer mentions sacrifice “of bulls, of goats” in the Iliad and of “sleek black bulls to Poseidon, god of the sea-blue mane who shakes the earth,” in the Odyssey. The most common offering was the sheep, goat, or pig, but the ox and bull were also used, depending on the occasion. Animals were selected for their physical perfection, their horns gilded and adorned with ribbons and garlands. As the animal walked toward the altar, barley was thrown in its path to entice it and water sprinkled on its head, causing it to nod as in agreement with the proceedings. The crowd was silent, then sorrowful, acknowledging the sacrifice. The ritual turned into a feast, “while the people tasted the innards, burned the thighbones for the god” (Odyssey, Book III).

The contradictions inherent in religious sacrifice did not elude ritual participants, who ate little meat outside these religious feasts. The rituals may have expressed their uneasiness at killing animals for food and to appease the gods. Their ambivalence continued during the classical period, when even large domestic animals sustainable only in small numbers were used. “Our ancestors handed down to us the most powerful and prosperous community ... by performing the prescribed sacrifices,” wrote Athens orator Lysias, defending the practice. “It is therefore proper for us to offer the same ... if only for the sake of the success which has resulted from those rites” (*8*).

Moschophoros stuns for its ability to bring to life eons after its creation a moment of connection. The human face, wearing a smile, the single most appealing adornment then and now, is framed by the surrendered animal. The marble seems to melt in the calf’s unparalleled fragility and tenderness. Locked in a secure embrace, human and animal take a step together, an ear touching, a tail relaxed.

“My pawing over the ancients and semi-ancients,” wrote Ezra Pound, “has been one long struggle to find out what has been done, once for all, better than it can ever be done again, and to find out what remains for us to do” (*9*). Moschophoros captured the primeval ease between man and calf. What remains to do? For us the challenge is to get beyond the sacrifice. Whether to appease the gods or stop BSE, charred remains of cattle and other animals betray limited success in our symbiotic relationship. Increased animal translocation and ecologic transformation add to the intrigue, along with microbial changes now seen at the molecular level.

Moschophoros is not ancient. The statue exists in the present. It can be touched, viewed, and examined for universal meaning. Resilient and unchanged, it defies death. And like other marvels from antiquity, it takes the initiative in speaking to us. “The statues are not the ruins,” wrote Seferis. Inasmuch as we die before we step forward, “—we are the ruins” (*1*).
